# Explaining pre-emptive acclimation by linking information to plant phenotype

**DOI:** 10.1093/jxb/erab537

**Published:** 2021-12-16

**Authors:** Pedro J Aphalo, Victor O Sadras

**Affiliations:** Organismal and Evolutionary Biology Research Programme, Viikki Plant Science Centre, Faculty of Biological and Environmental Sciences, University of Helsinki, Finland; South Australian Research and Development Institute, and School of Agriculture, Food and Wine, The University of Adelaide, Australia; Forschungszentrum Jülich, Germany

**Keywords:** Adaptation, cues and signals, drought, eco-devo, epigenome, genome, information, phenome, preemptive acclimation

## Abstract

We review mechanisms for pre-emptive acclimation in plants and propose a conceptual model linking developmental and evolutionary ecology with the acquisition of information through sensing of cues and signals. The idea is that plants acquire much of the information in the environment not from individual cues and signals but instead from their joint multivariate properties such as correlations. If molecular signalling has evolved to extract such information, the joint multivariate properties of the environment must be encoded in the genome, epigenome, and phenome. We contend that multivariate complexity explains why extrapolating from experiments done in artificial contexts into natural or agricultural systems almost never works for characters under complex environmental regulation: biased relationships among the state variables in both time and space create a mismatch between the evolutionary history reflected in the genotype and the artificial growing conditions in which the phenotype is expressed. Our model can generate testable hypotheses bridging levels of organization. We describe the model and its theoretical bases, and discuss its implications. We illustrate the hypotheses that can be derived from the model in two cases of pre-emptive acclimation based on correlations in the environment: the shade avoidance response and acclimation to drought.

## Teaser

A model to explain the role of information acquisition, storage, and use at different time scales in the evolution and realization of pre-emptive acclimation is proposed and applied to examples.

## Introduction

### The importance of context and information in the study of plants

Current theory of the phenotype is lagging behind our fast-growing ability to generate genetic and phenotypic data (Noble, 2014; Sadras, 2021). We need conceptual models to explain and predict how these two types of data are causally interconnected, particularly for complex traits where an unjustified, unidirectional gene-to-phenotype model is implicitly still prevalent (Box 1, ‘Phenotype and downward causation’).

Box 1. **Key concepts and definitions**

**Abstraction, idealization, and effective theory**. ‘An abstract description of a system leaves a lot out. But it is not intended to say things that are literally false. An idealised description of a system is a description that fictionalizes in the service of simplification…’ (Godfrey-Smith, 2009). Effective theory allows modelling of the behaviour of the system without specifying all of the underlying causes that lead to system state changes; by definition, effective theories are agnostic to system mechanics (Flack, 2017); see also coarse graining.

**Coarse graining**. Coarse graining is a reduction of the microscopic details of a system. Plants sense individual aspects of the environment such as presence of neighbours and soil drying through reasonably well-established mechanisms, but the integration of presence of neighbours and dry soil remains a gap. The Flack (2017) model of coarse graining is an interesting perspective for such integration. In this scheme, *E* are environmental states including presence of neighbours and dry soil, and *P*(*i* =neighbour) and *P*(*i* = dry soil) are the respective algorithms by which these components, *i*, in *M* estimate environmental states. *C* is collective computation by *M*_*i*_ of *S*, the macroscopic variable, and *D* is the downward causation via *i* in *M* reading *S* and tuning the phenotype to the integrated condition of presence of neighbours and dry soil. Contemporary efforts in quantifying collectivity (Daniels *et al.*, 2016) could provide novel insights into plant integration of multiple cues and signals.

**Cue and signal**. Karban (2015) writes ‘…I will regard responses to stimuli as examples of plants sensing cues but not communicating.’ In the case of ‘signal’, definitions vary among authors, but in general criteria are stricter than for ‘cue’, in many cases implying communication that is beneficial to both parties involved, and that emission and sensing of the signal have evolved for the purpose of sharing information. In practice, a clear distinction between cues and signals for specific interactions is difficult (see Karban, 2015, chapter 1): signals are thought to be sent while cues happen, a distinction that in the case of plants we can only guess from the observed behaviour.

**Decision making**. We use this term as an abstraction indicating a ‘choice’ of one out of many possible development ‘paths’ available to an organism. By this, we do not imply that plants make conscious choices, or that consciousness might play a role in the model we present (see Taiz *et al.*, 2019).

**Information**. The role of information we discuss here is related to an organism’s interaction with its environment. Our model is agnostic about considering the process of evolution itself as a flow of information or not (see Godfrey-Smith, 2013, chapter 9), which is not required to be able to consider DNA as a memory of past evolutionary events.

**Maternal effects**. Maternal effect is ‘the causal influence of the maternal genotype or phenotype on the offspring phenotype’ (Wolf and Wade, 2009), and the continuity of the phenotype refers to the ‘unbroken and overlapping connections between the generations mediated by parentally constructed offspring phenotypes (e.g. eggs, spores, seeds)’ (West-Eberhard, 2003). The seed thus carries information across generations. In organisms with no parental care, such as plants, maternal effects can be attributed to two mechanisms: offspring provisioning and epigenetics. Maternal offspring provisioning has a quantitative component, (i.e. seed mass reflecting the amount of reserves and embryo size), and the transmission of somatic or cytoplasmic factors mediated by nutrition and metabolism (Kuijper and Johnstone, 2015); some plants can also transmit microbial symbionts to the progeny, which can influence offspring fitness (Gundel *et al.*, 2017). Epigenetics—a change in gene expression without base sequence alteration—involves processes such as DNA methylation, RNA-directed DNA methylation, nucleosome histone post-translational modifications, and regulation of small RNA activity; some of these modifications are stable and form the basis of ‘stress memory’ that is carried over across generations (Springer, 2013).

**Memory, behaviour, and problem solving**. Broadly speaking, memory is the storage of information that has been acquired through sensing of cues and/or signals. Behaviour is used in different contexts, such as psychology and mathematics, and in the second case describes the general properties of outputs given certain inputs. In this second sense is that we consider behaviour applicable to plants and the outcome of sensing of cues. Through idealization, some of this behaviour may be explained as contributing to solve a ‘problem’ faced by an organism.

**Model**. According to Fisher (1930, p. ix) ‘The ordinary mathematical procedure in dealing with any actual problem is, after abstracting what are believed to be the essential elements of the problem, to consider it as one of a system of possibilities infinitely wider than the actual, the essential relations of which may be apprehended by generalised reasoning…, which may be applied at will to any particular case considered.’ Here we do not attempt a mathematical formulation of our model, although this might be possible in the future.

**Noise**. Noise usually refers to disruptions that interfere with the transmission or interpretation of information. However, there are more nuanced aspects to noise. Weinstein and Pavlic (2017) note at least two functionally beneficial aspects of noise. One is noise as a source of variation whereby isogenic populations can vary phenotypically due to variation in gene expression. The second is the role of noise in non-linear systems, particularly those with one or more thresholds for which a small variation in input gives rise to disproportionate differences in output, illustrated by large shifts in global climate in response to small changes in insolation. Krakauer (2017) emphasizes that biological units (cells, organisms, populations) with accurate information relevant to fitness, ‘endeavour to keep this information to themselves and share informative signals only with those with whom they have found means to cooperate’. He makes the case for living phenomena as evolutionary cryptosystems, and interprets the c-value paradox (i.e. lack of correlation between genome size and phenotype) and junk DNA in the light of this theory.

**Phenotype and downward causation**. The phenotype includes all traits of an organism other than its genome (West-Eberhard, 2003). Downward causation (teal green arrows in Fig. 3) refers to the causal influence of higher levels of organization on lower levels of organization (Noble, 2012; Flack, 2017). There are ~30 cell types in a typical plant and ~120 cell types in vertebrates.

Thus, in contrast to the unidirectional arrow from genotype to phenotype in the central dogma of molecular biology, developmental biology highlights the diversity of cellular phenotypes derived from a single genome, and the importance of phenotype-driven differential gene expression (West-Eberhard, 2003; Noble, 2012). Mary-Jane West-Eberhard’s theory of phenotypic development and evolution emphasizes that ‘the individual’s genotype can never be said to control development. Development depends at every step on the pre-existent structure of the phenotype, a structure that is complexly determined by a long history of both genomic and environmental influences’. Meanwhile Noble (2012) states that ‘a difference in DNA sequence may have a wide variety of possible phenotypic effects, including no effect at all, until the boundary conditions are set, including the actions of many other genes, the metabolic and other states of the cell or organism, and the environment in which the organism exists’. The essence of the central dogma is that ‘coding’ between genes and proteins is one-way. As in Noble (2012), we favour the word ‘template’ to ‘coding’ since ‘coding’ already implies a program.

Context, as used in this review, includes the environments to which an individual organism and its ancestors have been exposed, and is key to understanding development, behaviour, growth, and reproduction. The importance of context stems from the non-additive nature of the influence of its components onto plant responses. However, context is often overlooked in the design of experiments and in the interpretation of the plant phenotype, for example when gas exchange measured in individual leaves ignores the effects of both leaf and canopy boundary layers (Jarvis and McNaughton, 1986), or when metabolic profiles of plants ignore the artefacts associated with step changes in irradiance compared with the day–night sinusoidal irradiance regime or irregular variation due to clouds (Annunziata *et al.*, 2017), or when interference between adjacent maize plants in a greenhouse is ignored (Chen *et al.*, 2019).

The importance of context varies. For constitutive traits, biotechnology applied to crop protection has been very successful, as illustrated in the reduced reliance on broad-spectrum insecticides for cotton and maize crops transformed to express Bt (*Bacillus thuringiensis*) toxins targeting lepidopteran pests (Fitt, 1994; Downes *et al.*, 2016), and herbicide resistance in soybean favouring no-till systems (Viglizzo *et al.*, 2011; Marinho *et al.*, 2014). In contrast, for traits under complex regulation and naturally part of acclimation responses, biotechnology has underdelivered, as illustrated by meagre success in improving crop yield despite significant efforts (Passioura, 2006, 2020; Tardieu, 2012; Gilbert, 2016; Dalal *et al.*, 2017).

Gene expression, development, growth, resource allocation, and yield depend on stand density and genetic identity of neighbouring individuals, hence the importance of plant–plant interactions, which are part of the context for both wild species and crops (Geisler *et al.*, 2012; Crepy and Casal, 2014; Bowsher *et al.*, 2017; Murphy *et al.*, 2017a, b). Competition for resources among plants depends directly on the acquisition of resources and indirectly on the acquisition of information allowing prediction of future contests for resources (Ballaré *et al.*, 1987; Novoplansky *et al.*, 1990; Aphalo and Ballaré, 1995; Aphalo *et al.*, 1999). Thus, competitive behaviour as elicited by perception of signals and cues has temporal and rate-related constraints dependent on both a plant’s stage of development and size and those of its neighbours (Novoplansky, 2009).

For crops, yield does not normally scale from single plant to stand (Pedró *et al.*, 2012), and for natural vegetation, distribution of plant species in most cases cannot be predicted from survival of plants growing in isolation. Although neighbours are in both cases important, there are differences between wild plants and crops in their responses to them as nature selected for but agriculture selected against competitive ability (Denison, 2012; Weiner *et al.*, 2017; Weiner, 2019; Cossani and Sadras, 2021). In addition, compared with crop stands, natural vegetation is often more diverse, leading to more complex interactions. Although context has been considered in many vegetation and ecosystem studies, our understanding of the role played by plants’ multiple sensory mechanisms and informational signalling in fitness is only partial and mostly qualitative.

Many traits of ecological or agronomic relevance including fitness and grain yield result from the interaction of numerous cellular signalling pathways modulated by perceived cues and signals (Box 1, ‘Cues and signals’). For these traits, fine-tuned regulation is more important than overall metabolic capacity. Both fitness acquired through evolution and improved crop yield depend on the orchestration of the regulation of multiple developmental, morphological, physiological, and molecular characters including many not directly related to the acquisition of energy and matter (West-Eberhard, 2003).

Earlier we have argued that to understand plant–plant interactions it is not enough to consider resources because the ability of a plant to acquire these resources depends strongly on its ability to acquire and use information (Aphalo and Ballaré, 1995). This view has been supported by later research and has been influential in the development of an approach to the study of plants based on the concepts of behaviour and ‘problem solving’ (Trewavas, 2009). Twenty-five years later, here we present a conceptual model that expands the scheme of Aphalo and Ballaré (1995) by connecting the properties of the environmental context, natural selection, molecular signalling, and genetic and epigenetic mechanisms using an information-based view.

Our approach is inspired in sensory ecology and biosemiotics. Sensory ecology is a key aspect of the study of animal life (Dusenbery, 1992; Stevens, 2013). Biosemiotics, following Sharov (2016), emphasizes dynamic aspects of signs at the evolutionary and developmental time scales, featuring ‘constructivism’ in the sense that ‘…everything has to be constructed: sense organs—to detect signals; networks—to integrate and analyse signals; effector organs—to respond; memory—to store information; subagents—to perform downstream tasks including lower level construction; body—to integrate all functional units; niche—to live in; tools and resources—to increase functional efficiency; and signs—to support communication between parts of an organism and with other organisms’.

When reviewing the evolution of responses to stressors from the perspective of animal development, Badyaev (2005) stated that ‘When a stressor is reliably preceded by other environmental changes, their mutual recurrence facilitates the establishment of stressor recognition, assessment and avoidance strategies, such that an evolved stress-specific strategy does not involve an activation of an organism-wide stress response’. Novoplansky (2016) wrote that ‘[plants can] perceive, integrate and adaptively respond to myriad internal and external cues that are correlated with their future environments, in ways that maximize their life-time performance.’ Consequently, stress reactions are triggered when there is a discordance between the environment previously experienced and the current one (Badyaev, 2005); that is, when suitable anticipatory responses are not triggered in time to be effective or have not evolved. Evolution of anticipatory responses depends on the availability of suitable cues and signals. Bet-hedging is an alternative strategy that moderates risk of catastrophic failure even when the time course of environmental variation cannot be reliably anticipated (Childs *et al.*, 2010; Shemesh and Novoplansky, 2013). Bet-hedging can, for example, be implemented as a constitutive tolerance response to a stressor that appears as a ‘waste’ of resources in the absence of the stressor.

In this review we use the terms ‘decision’, ‘memory’, and ‘behaviour’ for plants only to refer to an abstract functional role, with no reference to biological implementation and without implying volition or consciousness (Box 1, ‘Decision making’ and ‘Memory, behaviour, and problem solving’). As Kauffman (2016) states:

‘…*E. coli* must ‘sense’ its world and has done so by evolving receptors for many signals, from glucose to acidity…This sensing of its world’s possible states, as given, for example, by the bound and unbound states of receptors for glucose, hydrogen ions, and so on, constitutes ‘biosemiotics’ at its root. Once life exists, sensing of its world was of selective advantage. But given that sensing, the *E. coli* must ‘evaluate’ ‘good for me and bad for me’, it must make a ‘decision’ to approach food or flee toxin, and then it must be able to act in the world to achieve an instrumental ought. Once doing exists, so do instrumental, not yet ethical ‘oughts’…’

In a theoretical analysis of the control mechanisms of annual cycles in vertebrates, Wingfield (2008) discussed the role of acclimation and fitness in variable environments. Wingfield’s framework includes five categories of cues, which are relevant to account for environmental influences on the growth and yield of cereals (Sadras and Slafer 2012): (i) developmental cues (e.g. tissue interactions); (ii) initial predictive information including environmental cues that allow long-term predictions (e.g. photoperiod); (iii) local predictive information allowing fine-tuning (e.g. rainfall or temperature); (iv) synchronizing and integrating information [e.g. social stimuli, red (R):far-red (FR) photon ratios in plant canopies]; and (v) labile perturbating factors (i.e. unpredictable environmental events).

Donaldson-Matasci *et al.* (2013) analysed the implications of environmental variability in cues used by organisms for predictive acclimation, and Novoplansky (2016) discussed anticipation in plants using the term ‘future perception’ to describe what we will call here biological forecasting. We prefer biological forecasting as this term better highlights the role of uncertainty in perception-based temporal extrapolation by organisms.

Resilience of ecosystems is the result of events at multiple levels of biological organization (Thorogood *et al.*, 2020, Preprint) of which here we consider the evolution and function of anticipatory plasticity in plants. We propose a conceptual model that links developmental biology and evolutionary ecology with the acquisition of information by the sensing of cues and signals. The model is based on the idea that the plant ‘reads’ much of the information in the environment not from individual cues and signals but instead from their joint multivariate properties such as temporal and spatial correlations. Our model can be used to generate testable hypotheses at different levels of organization. Here we describe the model and its theoretical bases, and illustrate the hypotheses that can be derived from it. We apply the model to a well-understood case of pre-emptive acclimation in plants, the shade avoidance syndrome, and an additional case for which we hypothesize an information-dependent mechanism: pre-emptive acclimation to drought upon exposure of plants to UV radiation.

## Information acquisition and use

Plants have numerous sensory systems capable of perceiving variation in the environment with high resolution (see Karban, 2015). New, unexpected senses have been described or postulated for plants, such as perception of magnetic (Ahmad *et al.*, 2007; Maffei, 2014) and electrical fields (Hebbar and Sinha, 2002), sound (Gagliano *et al.*, 2012) and mechanical vibration or contact (de Wit *et al.*, 2012), and discrimination among volatile molecules or cocktails of volatile molecules (Pierik *et al.*, 2014). Plants can communicate with each other and with other organisms using different signals (Falik *et al.*, 2012, 2014; Pierik *et al.*, 2014). Plants also utilize delayed responses, after-effects or ‘memory’, and spatial and temporal averaging (Sung and Amasino, 2006; Bruce *et al.*, 2007). The capabilities for self-recognition (Gruntman and Novoplansky, 2004) and kin recognition (Crepy and Casal, 2014; Bowsher *et al.*, 2017; Murphy *et al.*, 2017a, b) have also been described. Kinases play a central role in perception and signalling in plants (e.g. Osakabe *et al.*, 2013; Bourdais *et al.*, 2015). It is noteworthy that kinases—key enzymes in cellular signalling—are more abundant in plants than in animals (Idänheimo, 2015), suggesting that metabolic signalling could, from the point of view of information processing, partly substitute for the lack of a nervous system in plants (Idänheimo, 2015). Furthermore, capacity for perception and response to signals and cues does not pre-suppose consciousness or intelligence in plants (Taiz *et al.*, 2019).

Acclimation involves ‘decisions’ (*sensu*Kauffman 2016) about development, morphology, chemical composition, and physiology. Mechanistically, most often the first committed responses are changes in the expression of genes upstream of signalling cascades that can result in some cases in profound changes in metabolic pathways, plant morphology, and behaviour. For example, in the annual cycle of trees, several informational signals and their memories are a source of information for the timing of phenology and the modification of metabolism and cellular components leading to cold hardiness (Hänninen and Tanino, 2011; Hänninen, 2016).

We define ‘normal acclimation’ as a response to a gradual increase of the strength of the stressor, or repeated exposure to the stressor, while we define ‘pre-emptive acclimation’ as acclimation triggered by sensing of cues or signals that usually precede exposure to the stressor. There are several well-documented examples of pre-emptive responses by plants in addition to the example in the preceding paragraph: (i) to future shading (Ballaré *et al.*, 1987; Novoplansky *et al.*, 1990); (ii) to changing nutrient availability in the soil (Shemesh *et al.*, 2010, 2011); (iii) to impending drought (Falik *et al.*, 2012; Robson *et al.*, 2015); and (iv) to high risk of an imminent attack by herbivores (Ballaré, 2009; Karban, 2015). The complementary idea of acclimation to favourable conditions is equally true, as considering a given condition as positive or negative depends on what, we as observers, choose as the ‘normal’ reference condition, for example the photoperiodic modulation of mortality of florets in the ear of the wheat plant, whereby day length acts as a cue that anticipates the duration of grain filling (Ghiglione *et al.*, 2008).

**Fig. 1.**
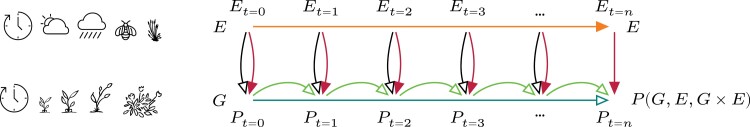
Pre-emptive acclimation and selection: orange, time course of one realization of the environment (*E*) during the lifetime of an individual of a genotype (*G*); teal, time course of phenotype (*P*) through development, growth, and acclimation; black, sensing of cues and signals targeted and initiated by the plant, leading to acquisition of information; red, selective pressure from the environment; green, (time-consuming) acclimation response. The phenotype is the outcome of the expression of the genotype in an environment, *P*(*G*, *E*, *G*×*E*), where *G*×*E* describes the non-additive interaction. Filled arrowheads indicate direct dependence on the environment, while open arrowheads indicate dependence mediated by the genotype and phenotype. For simplicity, we plot continuous time as discrete steps.

Acclimation of plants to stress, by definition, precedes the stress it helps tolerate or avoid. This follows from the definition of acclimation as a process that requires time and is rarely fully reversible. Within the life of an individual, its acclimation takes places concurrently with exposure to the environment, but with a lag. Fitness is determined by the dynamic interaction between genotype and environment through the life cycle (Fig. 1). This interaction involves acquisition of information by sensing cues and signals, and environmental and developmental constraints. This process repeats for each individual during each generation, driving evolution, including the evolution of pre-emptive acclimation.

Our analysis focuses on information, rather than on physiological mechanisms or ‘implementation’. This is a more abstract viewpoint, which favours generalization at the expense of mechanistic descriptions of individual cases (Box 1, ‘Abstraction and effective theory’). The difference between the usual metabolic signalling diagrams and an information-based model is that the abstractions are based on different criteria, suitable for the analysis of different types of questions: those related to proximal mechanisms and those enquiring about ultimate evolutionary causes.

In addition, when studying acclimation and adaptation, we are concerned with the performance of whole plants. Consequently, even when dealing with mechanism, it is best to study responses as syndromes affecting whole individuals rather than responses of isolated processes or features (Aphalo, 2010; Pierik and Testerink, 2014). By doing so, we will be able to capture interactions among the individual responding processes and their role in the behaviour and performance of whole plants in communities (Donald, 1963; Harper, 1977).

When we ask questions related to fitness and evolution, the plant’s environment needs to be included as a component of the system under study. Pierik *et al.* (2014) have highlighted the need to take into account the community in which the plants grow; here we add the abiotic environment and, most importantly, the statistical relationships among the various biotic and abiotic variables. However, as Stevens (2013) emphasized for animals, we should do this with reference to the sensory abilities of each species. Most research of plants’ sensory capabilities has centred on the plant and its responses rather than on describing the multivariate dynamics of the plants’ environment. Existing studies are few and frequently limited to the dynamics of aggregate summary variables (e.g. R:FR photon ratio versus herbaceous canopy development, Evers *et al.*, 2006), or long-term dynamics (e.g. species succession and seasons in forests, Ross *et al.*, 1986).

## The non-random components of environmental variation

Patterns of temporal fluctuation in physical and biological phenomena and their predictability play an important role in ecology and evolution, and can be analysed using statistical methods for time series (Colwell, 1974). Colwell (1974) used the terms constancy and contingency to name the sources of predictability. Since the 1970s, the analysis of time series has developed, extending its scope to include multivariate data as well as discrete events. The idea that temporal variation can be assigned to different generating mechanisms or processes and that these processes can contribute to predictability, remains valid.

To a large extent, variation in the environment has structure: variables do not vary independently of each other, or independently of their previous or future states. Hence, current and past states of variables can be a source of information for prediction of the future state of the same variable, the current state of different variables, or the future state of other variables. For any organism, predicting future conditions can be expected to contribute to fitness. Conditions include both normal events, which occur frequently, and infrequent extreme events— once over many generations. These uncommon events can impose limits to evolution (Gutschick and BassiriRad, 2003; Lyberger *et al.*, 2021).

From this it follows that within the constraints of the evolutionary process, and the reliability of available sources of information, most organisms, including plants, should be expected to acquire, store, process, and use information during their lifetime in decision making (Box 1, ‘Decision making’) related to acclimation. We should be aware, though, that predictability of events creates boundaries to the plastic behaviours that can persist in the long run versus bet-hedging strategies (e.g. Childs *et al.*, 2010; Grantham *et al.*, 2016). Natural selection of survivors to exceptional events may lead to behaviour that can be described as ‘risk aversion’ (Novoplansky, 2009).

Describing correlations and lags among environmental variables is crucial for understanding their role as sources of information for pre-emptive responses that depend on implicitly ‘forecasting’ future events. Autocorrelation describes correlation in time for a variable with itself; it is typical of gradual, cyclical, or repeating patterns of change. Cross-correlations describe the ‘parallel’ changes of two or more variables in time. If there is lag in a cross-correlation, it means that variation in one variable is consistently delayed compared with the variation in another variable, while both variables follow a similar pattern of temporal change.

**Fig. 2.**
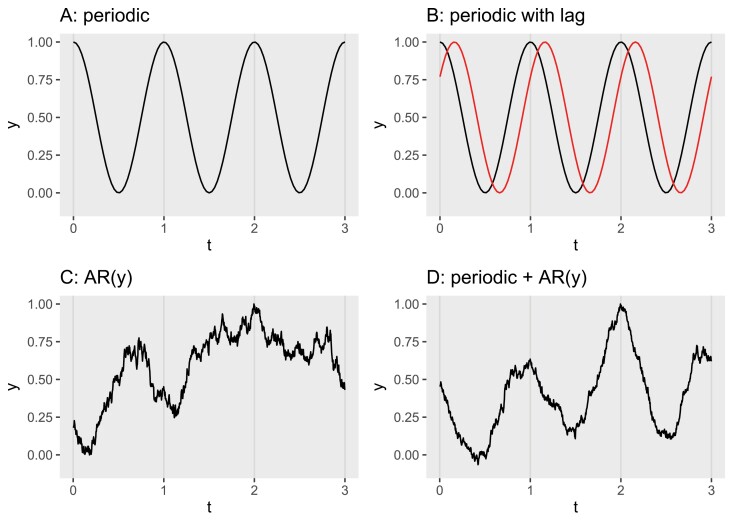
Artificial examples of patterns of environmental variation (*t* depicts time, and *y* the value of an arbitrary environmental variable). (A) Deterministic cyclic variation; (B) the same as (A) (in black), but adding a second variable with the same amplitude and cycle but lagged (in red); (C) an autocorrelated time series generated from a random process; (D) a combination of cyclic deterministic variation and autocorrelated ‘noise’ in the response, AR(*y*). See Box 2 for calculation details.

Some patterns of variation are both cyclic and deterministic, such as day length. In such a case, the future state of the variable can be predicted if the period, amplitude, and phase are known (see Fig. 2A for a simple example). Two such patterns can be shifted in time, and the early one directly used to predict the future state of the later one (Fig. 2B). Many patterns of environmental variation are not fully deterministic, but nonetheless are not completely random because of the presence of correlations. The simplest case for a time series is autocorrelation, in which values close in time are more similar than those further away in time. This kind of pattern can be simulated using random variation as a starting point (Fig. 2C). This demonstrates that information about the correlation acting on a random process is useful for forecasting the future state of a variable using its current or recent state as input.

Box 2. **Correlations in the environment**

Environmental variables are not independent and identically distributed (iid).

The state of individual variables is autocorrelated both in time and in space; for example, a warm day is more likely to be followed by another warm day than by a much colder day. The same is also true spatially, the soil water content 0.1 m away from the current location is more likely to be similar than that 1.0 m away. From a multivariate perspective, different environmental variables are correlated with each other; for example, within a single day, water vapour pressure in the air tends to vary little, but near noon when air temperature is higher, the vapour pressure deficit is usually at its maximum and relative humidity at its minimum. The mechanistic explanation behind these different correlations varies, but irrespective of their origin correlations carry information useful in forecasting. Information we also intuitively use in everyday life.

In Fig. 2 we show plots of time series artificially generated in R (R Core Team, 2021) assuming different generative processes. We describe here the algorithms used to generate each of the time series accompanied by brief explanations.

Figure 2A and B are the result of deterministic processes with cyclic variation with no random component. Based on arbitrary *t* values, representing an ordered sequence of distances in time or space from an origin, *y* values were computed without a lag as

$$\begin{array}{l} y_{i}=f(t_{i}), \end{array}$$

and with a lag as

$$\begin{array}{l} y_{i}=f(t_{i}+l), \end{array}$$

where *f* is a determinist cyclic function such as sin or cos and *l* is a lag (i.e. a constant shift along the *t* axis).

In the remaining panels, we use as a starting point a series of (pseudo)random values generated from the Normal distribution *N*(μ,σ). The series in Fig. 2C has no deterministic component, but it is generated by an autoregressive, AR(*y*), process where the value of *y* at the next time step *t*_*i*+1_ depends on a random component and the value of *y* at time *t*_*i*_. The series is generated recursively advancing one step of *t* at a time using R function diffinv() applied to a vector of independent and normally distributed values

$$\begin{array}{l} y_{i+1}=y_{i}+N{\left(\mu =0,\;\sigma =1\right)}_{i}. \end{array}$$

The series in Fig. 2D combines the deterministic cyclic component from Fig. 2A and the autoregressive random component of Fig. 2C.

In nature, these components jointly contribute to the observable variation such as cyclic, and random autoregressive (Fig. 2D). These latter examples are presented for a single variable for simplicity, but correlation among ‘noisy’ variables can also provide useful information for the prediction of the future state of lagged variables. Until now, we have centred the discussion on changes in the time domain. Similar correlations exist in the spatial domain. In certain cases, lag in time is caused by differences in the speed of propagation in space. The temporal lag between two signals originating at the same point in space, but propagating at different speeds, depends on the distance travelled and their relative speeds.

It is important to realize that when such lags or correlations among variables are not part of the physical and chemical environment, organisms have the ability to ‘add’ signals to their environment that do have these properties. For example, the emission of plant volatile organic compounds (VOCs) in response to herbivory could generate a signal that propagates faster to neighbouring plants than the insects move, resulting in a delayed arrival of the insects with respect to the arrival of the VOC signal. In addition, as the activity of the herbivores triggers the emission of VOCs, the presence of VOCs in the air in the neighbourhood of a plant under attack is tightly correlated with the (impending) arrival of the herbivores. It must be stressed that here we are discussing correlations, and consequently the previous statement should be interpreted as the probability of insects soon reaching the target plant being higher when VOCs are present in the air than when they are not.

Superimposed on environmental patterns there is a significant amount of ‘random noise’ or variation to which we are unable to assign a deterministic origin. Statistics gives us the tools, as researchers, for separating interesting information from random variation or, so-called, noise (Box 1, ‘Noise’). Statistical algorithms can be computed in analogue systems as well as in digital ones, and it has been proposed that even primitive organisms can do ‘maths’ through metabolic signalling (Daniel *et al.*, 2013). As in the case of statistical time series analysis, different sampling and smoothing methods can be expected to play a role in information processing by organisms. Even sharing of information among neighbours may, in some cases, be equivalent to sampling and averaging over a larger area, which could be beneficial to all plants involved in the case of variables with dynamic spatial heterogeneity in their state, such as herbivory.

The needed ‘information processing’ can also be complex in the time domain because the timing of a response can be crucial for fitness. A cue such as night length is minimally affected by noise (Box 1, ‘Noise’) and consequently a very reliable source of information; even though night length is a reliable cue, its correspondence to seasons of the year is not monotonic: each night length occurs twice per year in opposite seasons. In contrast, daily temperature is affected by strong variation in its temporal course, with patterns changing year to year due to prevailing weather conditions. These differences in the quality of the information source lead to different strategies in its use. For reproductive induction by short nights, a single short night event can inform about seasonal timing—leading to experimental observations of a single night break inducing flowering in some species (Jackson and Thomas, 1999). In contrast, temperature requirements for developmental events are most frequently a combination of previous ‘accumulated’ low or high temperatures and current temperatures (Baulcombe and Dean, 2014). An example of the use of multiple cues, functioning on a shorter time scale, is the complex interplay of cues perceived through different photoreceptors (Casal, 2013; Rai *et al.*, 2021) that also includes the temporal integration of these cues through the day, which apparently prevents a premature, or too strong, shade avoidance response under moderate shade (Casal, 2012; Sellaro *et al.*, 2012). In other cases, redundant sources of information can substitute for each other: for seed germination in many species, the well-known ability of alternating day/night temperatures to substitute for or modulate a high R:FR photon ratio requirement can be thought as having a partly overlapping role in the detection of bare (unshaded) and own-depth-in-the-soil for seeds (Benech Arnold *et al.*, 1988; Vazquez-Yanes and Orozco-Segovia, 1994). This redundancy, possibly stemming from the dual role of phytochromes as light and temperature sensors (Casal and Balasubramanian, 2019), can be thought as reflecting an overlap in information content between two environmental cues. Both qualitative and quantitative cues may provide information, but the adaptive advantage of responses to cues depends on the local environment as a whole, leading to broad genetic variability in natural populations (see Murfet, 1977). For example, in addition to the well-known correlation of photoperiodic responses of plants to seasonal variation in temperature, similar correlations to the local timing of the rainy season have been described (Murfet, 1977; Ryan *et al.*, 2016).

The more and better information is available—including on the context—the more reliable forecasts tend to be (Hyndman and Athanasopoulos, 2018). What we know about plants indicates that the regulation of metabolism and development relies on multiple sources of information combined through complex signalling networks containing multiple feedback loops and points of interaction (Ballaré and Pierik, 2017; Rai *et al.*, 2021). This is at the core of why extrapolating the results of experiments done in an artificial context into natural or agricultural systems almost never works for characters whose environmental regulation is important for the organism’s fitness: biased relationships among the states of different variables in both time and space may disturb the information decoded by the plant, returning ‘accidental’ phenotypes (e.g. Annunziata *et al.*, 2017) due to a mismatch between the selection history reflected in the genotype and the artificial growing conditions guiding its expression into phenotype. In addition, at the metabolic and signalling level, organisms have redundant paths for regulation, and compensatory regulation may mask the effect of altering one or few components (Ovaska *et al.*, 1992; West-Eberhard, 2003; Noble, 2012). Inconsistent results under controlled and natural environments are common, and are a bottleneck for the directional biotech pipeline from lab to field (Chan *et al.*, 2020). One striking example is that of the UV-B photoreceptor UVR8 in Arabidopsis: UVR8 dysfunction was reported as highly detrimental to growth in a unique sun simulator chamber designed to simulate the natural radiation environment (Favory *et al.*, 2009). However, that *uvr8* mutants can survive and flower in sunlight (Morales *et al.*, 2013) and grow normally in the same growth chamber under a slightly different illumination regime suggests that small differences in the timing of UV-B exposure within the photoperiod are important for tolerance (Rai *et al.*, 2019). Only considering the spectral properties of sunlight together with the spectral and photochemical properties of the UVR8 photoreceptor has allowed an understanding of how plants perceive solar UV radiation (Rai *et al.*, 2021).

Life history, development, allocation, morphology, and physiology adapt and acclimate in coordination, and in the case of crops contribute to yield. For example, theoretically it should be possible to improve the energy conversion efficiency of the C_3_ metabolism in plants (reviewed by Raines, 2011; Evans, 2013; Furbank *et al.*, 2015; Reynolds *et al.*, 2021). However, a lack of understanding of how and why such apparent inefficiencies may contribute to overall plant fitness makes setting physiological targets for crop breeding extremely difficult (Denison, 2015). The complexity of metabolic interactions, trade-offs between traits, issues of scale and levels of organization, and environmental factors over-riding genetic variation converge to constrain the opportunities for breeding and selection for higher photosynthesis (Denison, 2012; Sadras and Richards, 2014; Furbank *et al.*, 2015; Sinclair *et al.*, 2019). Similarly, genetic modification targeting improved drought tolerance in crops has rarely been successful (but see González *et al.*, 2019), while traditional breeding has allowed a sustained improvement of yield in dry environments for many decades (Sadras and Richards, 2014; Passioura, 2020).

The current poor record of success does not mean that indirect, trait- or genetics-based, attempts at crop improvement are inherently of little use. Instead, it shows that the dominant conceptual model of crop phenotype has been misconstrued or oversimplified; it has, among other things, failed to account for traits related to acclimation, which depend on signalling networks and coordination of multiple responses that capture the complexity of environmental variation.

## Strategies

According to DeWitt and Langerhans (2004), plants have evolved four contrasting strategies in response to environmental variation: (i) specialization, whereby a single phenotype is produced that is well adapted to a particular environment even though the specialist may experience a range of environments; (ii) generalization, whereby a ‘general purpose’ phenotype is produced, with moderate fitness in most environments; (iii) bet-hedging, whereby an organism produces either several phenotypes (e.g. among units in a modular plant, such as co-existence of juvenile and adult leaves in some trees) or single phenotypes probabilistically (e.g. morphological and dormancy polymorphism in seeds produced by an individual plant); and (iv) phenotypic plasticity, whereby alternative phenotypes are produced in response to environmental cues. Modelling these four strategies under the assumption of perfect phenotypic plasticity and a simplified range of environments returned a ratio of fitness after four generations of 1:1.6:1.5:25 (DeWitt and Langerhans, 2004). The conclusions from this type of analysis are that in the absence of constraints, unrestricted plasticity is superior in variable environments, and the fact that unrestricted plasticity is not ubiquitous suggests the existence of ubiquitous constraints. The more likely constraints include a relatively high cost for plasticity, developmental constraints, and unreliability of environmental cues that guide development (DeWitt and Langerhans, 2004; Sadras and Slafer, 2012; Murren *et al.*, 2015).

A given phenotype can follow different strategies in relation to different features of its environment and, in addition, the degree of phenotypic plasticity can concurrently differ between plant traits. A genotype may express a trait that is very responsive to environmental cues, such as internode elongation versus R:FR photon ratio, but less responsive to other traits. Although the degree of plasticity is trait dependent, evidence supports partial rather than full independence between the genetics of a trait (e.g. phenology or grain weight) and the genetics of the trait’s plastic response to the environment (Reymond *et al.*, 2003; Lacaze *et al.*, 2009; Marguerit *et al.*, 2012; Alvarez-Prado *et al.*, 2014; Sadras *et al.*, 2016), as anticipated by Bradshaw in the 1960s (Bradshaw, 1965). An important consequence of the partial independence in the genetic control of plasticity and the trait *per se* is that plasticity can evolve independently of the trait (David *et al.*, 2004; Pigliucci, 2005; King and Roff, 2010). Novoplansky (2009) discussed the implications of plasticity itself being plastic, using the term metaplasticity while emphasizing risk management and plant–plant interactions.

A less frequently discussed aspect of these strategies is that many morphological and developmental responses of plants are slow compared with the speed of change in availability of resources. Moreover, such responses depend on the use of photosynthates, mineral nutrients, and other resources of limited availability. Consequently ‘valuable’ resources need to be invested, which may be recovered for re-use only at a very significant ‘loss’ (Bloom *et al.*, 1985). For example, benefits to plants from responding to current light quality cues may depend on forecasting, or anticipating, how much and how fast neighbours will grow (Novoplansky, 1991).

## Decision making

The use of economic models as an analogy for describing regulation of metabolism, capture, and allocation of resources has a long tradition in biology in general (Ghiselin, 2000) and plant ecology (Bloom *et al.*, 1985). Here we highlight a specific aspect of this analogy, which has not been previously used in plant research: the analogy between the use of information and forecasting tools in dynamic resource allocation in human enterprises and the equivalent dynamic regulation of investment of limited resources by plants. Keeping this analogy in mind while reading the rest of this review is important for understanding the logic behind our conceptual model.

Acclimation, as a form of investment, can be based on continuous dynamic adjustment of allocation, for example growth allocation to shoots versus roots, or on a switch-like choice of a developmental programme, such as a switch from vegetative to reproductive stage. Reality has more nuances but, as a working classification, acclimation and development decisions can be considered as discrete alternatives or the value on a continuous scale used as set points of a feedback or feed-forward control mechanism. West-Eberhard (2003) defines a switch point as ‘a point in time when some element of the phenotype changes from a default state, action, or pathway to an alternative one—it is activated, deactivated, altered, or moved.

Even if there are recognizable patterns, the stochastic component of the environment (Fig. 2) means that ‘acclimation-related decisions’ cannot be hard-wired. These decisions need to be taken ‘on-the-go’ during plant development and are subject to errors. This brings in the interplay of profit and risk. Different contexts, and different variables within a given context, will be subject to different amounts and types of variation. From the point of view of evolution, optimization of individual traits such as carbon acquisition or the use of water during photosynthesis cannot be thought as the ‘end target’ of natural selection or the best target for crop breeding (Sadras and Denison, 2016). We should expect risk avoidance to play a key role in long-term selection (Novoplansky, 2009). As plant species differ widely in their reproductive strategies and life histories, mechanisms for risk avoidance can also vary widely. For example, in plant species propagating mainly through seeds, completion of the life cycle, and successful reproduction in every generation could be thought of as mandatory for fitness (Amir and Cohen, 1990). However, mechanisms such as the maintenance of a large and long-lived seed bank in the soil can play the role of a ‘safety net’, allowing the survival of a population and its rapid recovery after exceptional catastrophic events.

As mentioned above, in some cases such as seed germination, decision making consists of a choice between discrete options, in this case binomial: to initiate growth of the individual as a whole or not. In other cases, it can be thought of as the adjustment of a set point on a continuous scale, for example the shoot:root ratio or the regulation of stomatal conductance. In this last example, it can even be thought of as a decision to change responsiveness. For example, long-term exposure to UV radiation can trigger a reduction in the response speed of stomata to a step increase in irradiance of specific colours of visible light (Aasamaa and Aphalo, 2016, 2017).

A parallel exists between these ideas and the management of limited resources by human enterprises. Decision makers use forecasting tools, based on statistics, in particular time series analysis, combined with information about the current market and economic situation to improve the long-term return from limited resources. One successful example is the management by power utilities of power generation and distribution capacity based on demand forecasting (Hyndman and Athanasopoulos, 2018). This parallel extends to other kinds of predictions (see Orrell, 2006; Kauffman, 2008), but we here emphasize the parallel between how organisms can achieve pre-emptive acclimation and statistical forecasting methods. If approached from a high level of abstraction, it can be seen that equivalent information sources and tools are used by human forecasters and organisms. The complex statistical models stored as computer programs and used for forecasting electricity demand in the above example, are equivalent to signalling networks and sensory mechanisms in an organism’s genome and used to ‘make favourable decisions’ on the use of limited resources frequently enough to allow both short-term fitness and long-term survival. The parameterized instances of these models could be thought of as equivalent to the genotype as expressed in different phenotypes.

Another parallel between the use of forecasting for resource allocation by human enterprises and organisms is that in both cases the context or environment is under directional change, for example technological progress and the availability of raw materials for economic markets versus evolution of other species and global change for organisms. This means that the criteria and models used in decision making need to evolve, and their performance will also depend on the decision making by the rest of the community of managers as well as by other organisms in a biological community.

A further parallel, exceeding the scope of the present paper, is that consistency of decision criteria—embedded in similar predictive models—used by different traders and enhanced by reflexivity can exacerbate the risk of widespread financial losses (Beunza and Stark, 2012) while consistent responses among neighbouring plants can lead to excessive competition and even population collapse (i.e. in the absence of clear winners and losers, e.g. Yastrebov, 1996). Competition is detrimental to yield in crop stands of homogeneous genotypes with strong photomorphogenic responses (Boccalandro *et al.*, 2003; Pereira *et al.*, 2017; Wies and Maddonni, 2020). In both cases the combined behaviour of players driven by positive feedback—called resonance in Beunza and Stark’s text—can result in decisions that are bad for all players both individually and collectively, providing a further example of the importance of context.

These parallels allow us to borrow concepts and approaches used in statistical forecasting and to apply them to the development of a conceptual model for the functioning and evolution of pre-emptive acclimation in plants.

## Model

**Fig. 3.**
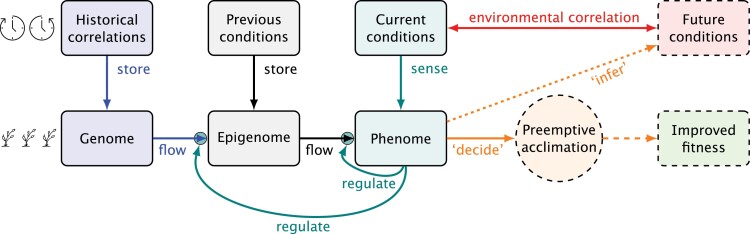
Flow of information in pre-emptive acclimation. Arrows represent flows of information: blue, retrieved from the genome (stored during evolution); black, acquired during an individual’s or its progenitor’s lifetime; teal, regulation of gene expression by phenome or downward causation; red, lagged correlation between two or more environmental variables; orange, outcome of information processing, which is a developmental ‘decision’ based on an implicit environmental forecast and with implications for fitness; green, future phenotype with ‘improved fitness’ relative, in probabilistic terms, to no acclimation. Dashed boxes and arrows represent the likely or forecasted future. Conditions refer to cues and signals both in the environment and in the plant’s internal status, corresponding to phenotypic plasticity, and developmental plasticity, respectively (West-Eberhard, 2003).

The model we present describes the use of information by organisms as a means of ‘deciding’ when and how to pre-emptively acclimate. If acclimation takes place before an organism is exposed to an event itself, either favourable or stressful, and this acclimation is triggered frequently before the actual event occurs, but only rarely when it does not occur, we can conclude that the organism has been able to forecast the occurrence of the event with a certain degree of success—with success defined as a pre-emptive response that increases fitness. As explained in the previous section, the parallel with statistical forecasting holds in many respects. In statistical forecasting, one possible approach is to use long-term time series data to develop a mathematical model, which is used together with recent and current data to forecast the future evolution of the demand for, for example, electrical power. Our model assumes a similar scheme for organisms, with the genome (viewed as a template for alternative development paths and behaviours) as the equivalent of the mathematical model of the data analyst, and the organisms’ sensory mechanisms and short-term memory as the equivalent of the short-term data acquisition and processing used by analysts in decision making (Fig. 3).

Our model is set at a high level of abstraction (Box 1, ‘Abstraction, idealization, and effective theory’) and provides the basis for theory. It considers information acquisition, storage, and use, without consideration of perception, transmission, storage, and processing mechanisms. It is an idealization in that we focus on information storage, flow, and use, and only consider acclimation to a single kind of future stress or favourable situation at a time. The novelty of our model is in explicitly taking into account simultaneously several possible sources of information and their joint statistical properties as inputs for decision making leading to pre-emptive acclimation in organisms.

We define three types of storage of information: genome, epigenome, and phenome, which span from evolutionary to intragenerational time scales. The mapping of these three stores of information onto chronological time thus depends on the life history of the organism.

We need to distinguish between maternal effects broadly understood and epigenetic regulation (Box 1, ‘Maternal effects’). The second is clearly a regulatory step involving mainly, if not only, information. We consider maternal effects dependent on resources (offspring provisioning), such as those associated with seed nutrient content or seed size, as part of the phenome. This distinction is coherent with the use of information as an abstraction.

The model assumes that as a consequence of natural selection, the use of different cues for acclimation is not necessarily related to cause and effect relationships in the environment. As long as a correlation exists that allows the organism to forecast a future event, evolution will favour the use of this cue as a source of information. From a statistical viewpoint, evolution generates a template for pre-emptive acclimation comparable with an empirical statistical forecasting model.

An important corollary is that the overall contribution of pre-emptive acclimation to fitness is not deterministic. Pre-emptive acclimation is a risk-taking game based on the probabilities and frequencies of occurrence of different events and the quantitative benefits and drawbacks from alternative patterns of capture and allocation of resources. All this is working within the boundary set by a probabilistic risk of population extinction—a binary response.

Our model integrates environmental factors to the extent that they are structured as described above. Further integration is beyond the scope of this paper, but coarse graining can be added in future versions (Box 1, ‘Coarse graining’).

## Example cases

To demonstrate the usefulness of our conceptual model for understanding the evolution of pre-emptive acclimation in plants, we will now describe two cases. One of them is the well-understood syndrome of shade avoidance, and the other is the poorly understood and controversial pre-emptive acclimation to drought mediated by plants’ exposure to solar UV radiation.

### Shade avoidance and pre-emptive acclimation

Shade represents for plants a restriction on the available photosynthetically active radiation (PAR), and in vegetation canopies shade is caused by neighbouring plants. The predominant strategy of sun-adapted plant species is to reduce this shading by increasing stem length and decreasing ramification, namely a shade avoidance syndrome (SAS). In plant canopies, low R:FR ratios are correlated with the presence of neighbouring plants that are alive (Smith, 1981), consequently plants can use the R:FR ratio as a source of information on the presence, size, and distance to neighbours. Furthermore, because FR radiation is not only transmitted but also reflected by plant leaves, the change in R:FR ratio starts well before any depletion in PAR. This time offset allows the triggering of the SAS before actual shading and contest for resources starts (Ballaré *et al.*, 1987).

**Fig. 4.**
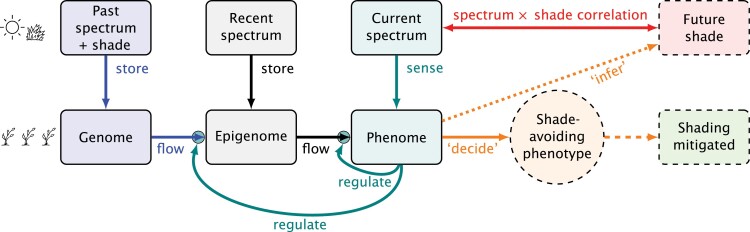
Flow of information in pre-emptive acclimation to shade by perception of radiation changes. Arrows represent flows of information: blue, retrieved from the genome (stored during earlier generations); black, acquired and/or ‘memorized’ during an individual’s or its progenitor’s lifetime; teal, regulation of gene expression by phenome or downward causation; red, lagged correlation between early changes in spectral irradiance and future low PAR irradiance; orange, outcome of information processing: a ‘decision’, based on an ‘implicit forecast of impending shade’, leading to developmental adjustments that would increase the probability of higher fitness in the presence of neighbours in comparison with phenotypes lacking pre-emptive acclimation; green, ‘shading mitigated’ compared, in probabilistic terms, with no acclimation. Dashed boxes and arrows represent the likely or forecasted future.

The ecology of responses to neighbours and shade mediated by perception of changes in spectral composition and irradiance was thought to be well understood after a long period of study (Holmes and Smith, 1977a, b; Smith, 1981; Deregibus *et al.*, 1983; Ballaré *et al.*, 1987); however, significant recent progress in understanding the physiological and molecular mechanisms (Casal, 2013) has been linked to identification of new ecological functions. Several recent publications have brought to light new and exciting details showing that plants are able to use much more than the R:FR photon ratio and blue irradiance as sources of information (Casal, 2013). Perception of UV radiation is also involved in acclimation to shade (Casal, 2013; Hayes *et al.*, 2014; Aasamaa and Aphalo, 2016; Moriconi *et al.*, 2018). A response to the blue:green photon ratio has been described as an additional cue of shade (Sellaro *et al.*, 2010). The same cues elicit different responses if received at different times of the day (Sellaro *et al.*, 2012), and temporal variability (i.e. sunflecks) affects responses compared with constant illumination (Sellaro *et al.*, 2011). Ethylene may be either a signal or a cue of the presence of neighbours in some environments (Pierik and de Wit, 2014). Physical contact could play a role when neighbours are growing very close together (Pierik and de Wit, 2014). The integration of the different cues is complex, and we lack an understanding of how the perception of neighbours works as an integrated whole (Pierik *et al.*, 2014; de Wit *et al.*, 2016; Ballaré and Pierik, 2017). In Fig. 4, the proposed model is applied to the flow of information involved in pre-emptive acclimation to shade. As we have considered together multiple cues of impending shade and ignored constraints, the model is a drastic simplification of reality. However, it allows us to derive useful testable hypotheses; for instance, (i) that light quality cues will trigger shade avoidance responses and (ii) that maternal effects on the readiness to respond to these cues will be relevant in species where seed dispersion is restricted to the neighbourhood of mother plants—when offspring are likely to grow in a very similar environment to mother plants.

### Soil drought and pre-emptive acclimation

Water availability is a major driver of ecosystem structure and function, regional patterns of land use, and global agricultural productivity (Ryan *et al.*, 2009; Chapin *et al.*, 2011; Stewart and Lal, 2018), hence the widespread interest in plant adaptation to drought (Morison *et al.*, 2008; Reynolds and Tuberosa, 2008; Kadam *et al.*, 2014). In the words of Tardieu (2012) ‘any trait or trait-related allele can confer drought tolerance: just design the right drought scenario’. This highlights the importance of context once again: tailoring adaptive traits to specific environments requires quantification of natural spatial, probabilistic drought patterns in terms of timing, intensity, and duration of water stress (Chenu, 2015). Going a step further, as discussed above, various cues and signals could function as sources of information for pre-emptive acclimation, adding further constraints to realistic drought scenarios. It has been shown that plant roots can perceive local soil drying before it affects the water status of a plant (Tardieu *et al.*, 1992; Wilkinson and Davies, 2010). This informs on the supply side of the water budget in relation to the soil volume already explored by the roots. The demand side of the water budget is described by evapotranspiration (ET), which for vegetation depends on reference or potential ET (ET_0_) and soil moisture (Monteith and Unsworth, 2008). In the absence of new precipitation, cumulative ET will determine the amount of water remaining in the soil at a future point in time.

**Fig. 5.**
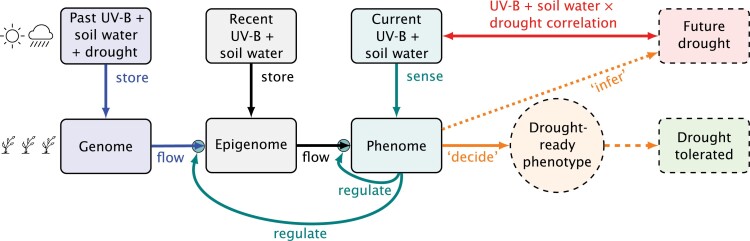
Information flow in pre-emptive acclimation to drought by perception of UV-B radiation and soil moisture. Arrows represent flows of information: blue, retrieved from the genome (stored during evolution); black, acquired during an individual’s or its progenitor’s lifetime; teal, regulation of gene expression by phenome or downward causation; red, lagged correlation between UV-B radiation and drought (e.g. low soil water content and high evaporative demand); orange, outcome of information processing: a ‘decision’, based on an ‘implicit forecast of impending drought’, leading to developmental adjustments that would increase the probability of higher fitness under drought in comparison with phenotypes with no pre-emptive acclimation; green, ‘drought tolerated’ compared, in probabilistic terms, with no acclimation. Dashed boxes and arrows represent the likely or forecasted future.

In this context, we ask how pre-emptive acclimation could help to improve fitness of wild plants and yield of crops under dry conditions. In this section, we use our generic model (Fig. 3) to describe a possible mechanism for the triggering of pre-emptive acclimation to drought by exposure to UV radiation (Fig. 5). We derive testable hypotheses and demonstrate using preliminary data how these hypotheses can be investigated. Before presenting the model, we justify why solar UV radiation is worthy of consideration in a context of multivariate correlations.

The interaction between UV-B exposure and drought tolerance, for plants growing outdoors, was first described in the context of stratospheric ozone depletion (Petropoulou *et al.*, 1995). Gitz and Liu-Gitz (2003) concluded that UV-B radiation could enhance drought tolerance in plants through photomorphogenic effects such as decreased leaf area, but added the caveat that drought tolerance could also result from strategies other than limiting water loss. More importantly, they highlighted the need to study the effect of UV-B exposure on the tolerance of drought stress by applying these treatments sequentially instead of concurrently as had been usual until then.

More generally, it has been suggested that perception of UV-B radiation through the UVR8 photoreceptor contributes to protection from various stressors (Hideg *et al.*, 2013; Singh *et al.*, 2014). In sunlight, because of the shape of the solar spectrum, UVR8 mediates the perception of both UV-B and UV-A2 radiation, namely solar radiation of wavelengths shorter than ~340 nm (Rai *et al.*, 2021). In an experiment comparing filters transmitting and attenuating solar UV radiation, we observed a strong effect, with near-ambient UV-B exposure preceding drought drastically enhancing drought tolerance in *Betula pendula* (Robson *et al.*, 2015). We have also observed acclimation of the speed of stomatal opening during a darkness to illumination transition as a result of exposure to solar UV radiation during growth, both in *Nothofagus obliqua* (Aasamaa and Aphalo, 2016) and in *Tilia cordata* (Aasamaa and Aphalo, 2017).

The finding that moderate UV exposure, perceived through the UV-B photoreceptor UVR8, acts as a regulator at the cellular level (Heijde and Ulm, 2012; Hideg *et al.*, 2013; Tilbrook *et al.*, 2013; Rai *et al.*, 2019, 2020) and that *Vicia faba* accessions from contrasting environments differ in their responses to same-generation and parental-generation exposure to UV radiation (Yan *et al.*, 2019, 2020) lend initial support to our hypothesis that physiological processes modulated by perception of a solar UV radiation cue could improve tolerance of future drought. Furthermore, an experiment with *Medicago truncatula* has shown that pre-exposure to solar UV-B+UV-A2 radiation suppressed the expression upon soil drying of most genes annotated as stress related that were expressed in plants not pre-exposed to solar UV-B+UV-A2 radiation (Yan, 2021).

In contrast to earlier views, we propose that UV radiation does not need to behave as an stressor to induce drought stress tolerance. UV exposure could play the role of a pure information carrier, triggering nonetheless pre-emptive acclimation to drought.

Figure 5 shows the flow of information involved in pre-emptive acclimation to drought. This is a simplification as we have ignored signalling among neighbouring plants—attributed to abscisic acid (ABA) in the soil (Falik *et al.*, 2011)—and the spatial heterogeneity of water availability, which can contribute to pre-emptive acclimation of neighbours of individuals experiencing drought first in a population. This model and the one presented above for the shade avoidance syndrome differ only in the labels, retaining exactly the same structure, which reveals that the generic model in Fig. 3 represents a framework suitable for the study of pre-emptive acclimation under different settings (see effective theory, Box 1).

We can derive three testable hypotheses from this model. (i) If UV exposure triggers pre-acclimation, and this response has evolved as a mechanism for enhancing tolerance of drought, a lagged environmental correlation must exist between solar UV exposure as perceived by plants and future water availability to inform about future drought. (ii) Responses triggered by UV-B+UV-A2 radiation will enhance future tolerance of drought through signalling mechanisms that can be traced to the perception of the cue. (iii) If UV-B and/or UV-A2 radiation function as a purely informational cue, rather than as a stressor, this cue must be perceived through a photoreceptor.

**Fig. 6.**
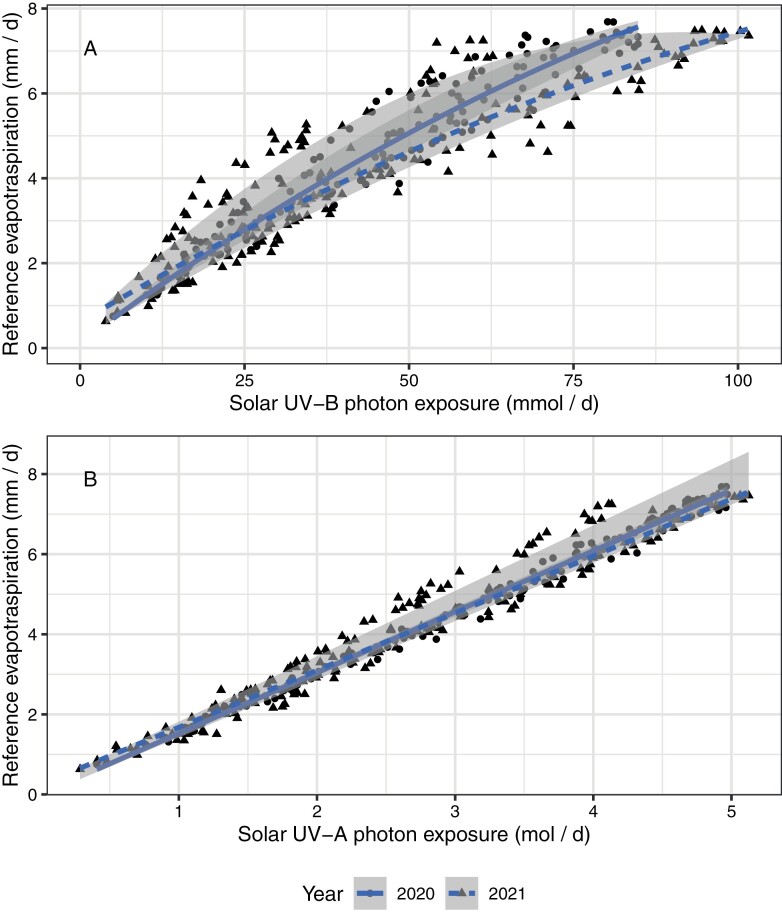
Day to day variation in solar UV radiation and reference evapotranspiration (ET_0_). Daily sums of estimated ET_0_ plotted against daily (A) UV-B and (B) UV-A photon exposures. Points indicate daily estimates from observations at 1 min intervals, lines depict the median regression, with grey shading indicating the quartiles (i.e. equivalent to the box in a box plot). Observations are for the growing seasons of years 2020–2021, at Helsinki, Finland (see Aphalo and Sadras, 2021, Preprint).

**Fig. 7.**
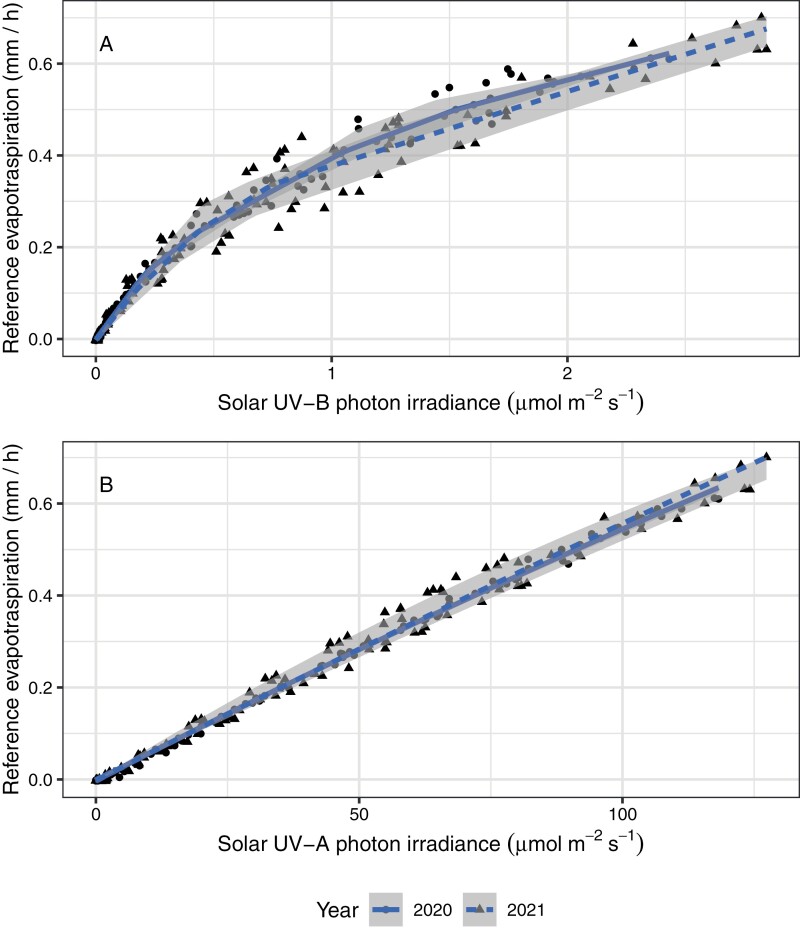
Variation in solar UV radiation and evapotranspiration through the photoperiod. Monthly means for each hour of the photoperiod of estimated reference evapotranspiration plotted against hourly mean (A) UV-B and (B) UV-A photon irradiances. Points indicate monthly estimates from observations at 1 min intervals, lines depict the median regression, with grey shading indicating the quartiles (i.e. equivalent to the box in a box plot). Observations are for the growing seasons of years 2020–2021, at Helsinki, Finland (see Aphalo and Sadras, 2021, Preprint).

To test hypothesis (i), which entails multivariate aspects of the environment, we looked for correlations between ET_0_ and different wavebands of sunlight using observations with very high temporal resolution for two growing seasons (Aphalo and Sadras, 2021). All bands of the solar spectrum when measured above the canopy are good predictors of ET_0_, including UV-B and UV-A radiation (Aphalo and Sadras, 2021). UV-A and UV-B radiation perform best at predicting variation within the photoperiod (Fig. 7), and longer wavelengths at predicting day to day variation in ET_0_ (Fig. 6). That solar irradiance and its components are good predictors agrees with the central role of the energy balance in evaporative demand and ET_0_ (Penman, 1948; Aphalo and Sadras, 2021). We concluded that UV-B exposure is an environmental cue carrying information useful for assessing the force driving ET. However, other regions of the solar spectrum carry similar information. Vapour pressure deficit and UV-B irradiance are also correlated within the course of the photoperiod (Aphalo and Sadras, 2021) as UV-B irradiance increases more with solar elevation than longer wavelengths. Even though the relationship between UV-B irradiance and evaporative demand is curvilinear, it can provide information about the demand side of the soil water balance equation. The relationship between actual ET and solar UV-B irradiance, and its consequences for soil moisture, remain to be analysed. A lagged correlation between solar UV exposure and soil water content should exist in dry spells between rainfall events as ET is a flux of water between soil and atmosphere. However, incomplete vegetation cover and low soil water availability frequently constrain ET to slower rates than ET_0_, probably making the correlations of solar UV exposure with ET and with soil water content weaker than between UV exposure and ET_0_.

Plants can acquire information on the supply side of their water budget, soil moisture, through their roots, with the hormone ABA being one of the within-plant signals of soil drying (e.g. Tardieu *et al.*, 1992; Wilkinson and Davies, 2010). Further, diffusion of ABA in soil is a signal with potential for plant–plant communication (Novoplansky, 2016), with a putative role in the coordinated regulation of water use among neighbouring plants required for efficient canopy water use (Aphalo, 1991). Taking into consideration that plant roots explore the soil to varying depths, a comprehensive analysis based on the profiles of root length density and soil moisture is needed to assess the relative importance of (i) ABA-mediated sensing of soil moisture, (ii) UV radiation-mediated sensing of evaporative demand, and (iii) integrating soil moisture and evaporative demand as cues for acclimation to future drought. Our model thus leads to the testable prediction that pre-emptive acclimation induced by exposure to solar UV radiation could involve ABA signalling.

Data from an experiment with Arabidopsis, involving exposure to solar UV radiation, but no drought treatment (Rai *et al.*, 2020), can be used to assess if solar UV radiation perceived through the UVR8 photoreceptor affects ABA metabolism and/or signalling. RNA sequencing after 6 h of exposure to different bands of the solar spectrum showed that the abundance of transcripts for several transcription factors responsive to drought or desiccation responded to UV-B and/or UV- A2 radiation in the wild type but not in a mutant lacking functional UVR8. Of these, the transcript abundance of AREB1 (other name ABF2, ABSCISIC ACID RESPONSIVE ELEMENTS-BINDING FACTOR 2) and of GBF3 (G-BOX BINDING FACTOR 3) was increased by exposure to solar UV-B while that of DREB1C (DEHYDRATION RESPONSE ELEMENT-BINDING PROTEIN) was decreased by UV-A2 radiation. For another transcription factor, ATHB7 (ARABIDOPSIS THALIANA HOMEOBOX 7), transcript abundance was decreased by exposure to UV-A2, but only in a null mutant lacking the UV-A1+blue light photoreceptors CRY1 and CRY2. ATHB7 is of special interest as it is also responsive to ABA and has similarity to HaHB4 (*Helianthus annuus* HomeoBox 4), which, as discussed below, when transferred to other crops confers enhanced drought tolerance under field conditions. These responses provide a link between solar UV radiation and the modulation of signalling dependent on ABA and drought.

On the other hand, the abundance of transcripts of DREB1A responded to UV-B radiation both in the wild type and in the UVR8 mutant, suggesting an additional signalling pathway independent of UVR8. However, interestingly, a motif analysis suggests that downstream regulation of expression of genes expected to bind to DREB1A depended on both UVR8 and CRYs. In contrast, neither changes in transcript abundance for genes involved in ABA metabolism nor changes in actual ABA concentration in leaves in response to solar UV radiation could be detected in the same experiment, while transcript abundance for a component of the degradation pathway of ABA, leading to phaseic acid, was responsive.

These results are consistent with the role of UV radiation-induced modulation of ABA signalling influencing readiness to acclimate to drought. Further studies are needed as a role for additional signalling mechanisms can be expected. For a full understanding, sequential measurements through the course of acclimation will be needed. It is also likely that both signalling and end responses differ between phenotypes adapted to different patterns of rainfall and/or evaporative demand (Schwinning and Ehleringer, 2001).

That exposure to solar UV radiation leads to changes in ABA-dependent signalling, a plant hormone which plays a key role in drought tolerance and signalling, supports hypothesis (ii) and that most of these changes require functional UVR8, supports hypothesis (iii). We can conclude that a non-stressful, sensory mechanism could enhance drought tolerance in response to solar UV exposure; in other words, an information-driven mechanism conceptually equivalent to anticipatory shade avoidance in response to changes in reflected FR light mediated by phytochromes. This is consistent with the current predominant view that for plants growing in sunlight, exposure to solar UV radiation is rarely a cause of stress (Jansen and Bornman, 2012).

In spite of this evidence for a sensory-driven link between exposure to solar UV radiation and drought tolerance, further experiments are needed to establish the mechanism(s) involved and their ubiquity in both cultivated and wild plants.

Whether further research will fully support or not our hypothesis about the informational role of solar UV radiation in pre-emptive acclimation to drought is not crucial here. The point is that applying our model to this difficult problem allowed us to generate useful and testable hypotheses applicable to both the expected response of plants and the properties of environmental cues. Based on this example, it is possible to imagine how our model will help in assembling the knowledge from different research fields into a broader and deeper understanding of plant phenotypes including pre-emptive acclimation.

## Discussion and implications

### On how to bridge the gap between laboratory and field

To profit from the mechanistic understanding obtained in controlled environments in natural and farming environments, we need to understand the ecological function of such mechanisms at an equivalent level of detail (Aphalo *et al.*, 2015). At both the mechanistic and ecological levels we need much more than to understand the structure and connections supporting signalling; we need to understand their function also at a higher level of abstraction based on information, taking into consideration both signalling and environmental cues.

If our proposed model holds for multiple cues, one major implication is that metabolic signalling interactions within an organism must reflect the environmental interactions present in the habitats where a species has evolved. Although the rooting volume in potted plants (Poorter *et al.*, 2012) and the spacing between plants growing individually in pots of equal volume and shape (Aphalo and Rikala, 2006) influence growth and morphology, using large pots set at a broad spacing does not solve this problem. Plants grow differently in controlled environments and outdoors (Poorter *et al.*, 2016), and the function of whole-plant canopies depends on responses of individual plants to light and other cues (Maddonni *et al.*, 2002; Pereira *et al.*, 2017). Consequently, full understanding of the role of metabolic signalling unavoidably requires taking into account the ‘normal’ growing environment of each species, even at the level of temporal and spatial variation and correlations among variables. We interpret this as a requirement for molecular and metabolic studies under field conditions, as recently discussed by Schuman and Baldwin (2018), even in the face of the frequently major practical difficulties involved. The gain is, of course, major, as such research will greatly enhance the practical usefulness of a vast amount of data acquired in controlled environments. However, this should not be thought as a competing approach, but as a complementary step, needed for making practical use under field conditions of our ‘how it is implemented’ understanding by developing a detailed understanding of ‘why such signalling or perception mechanism has evolved’ in wild plants and ‘why particular mechanisms have been retained, altered, or lost’ during domestication and breeding in crops. In all cases, quantitative probabilistic multivariate environmental characterizations are essential.

The contribution of pre-emptive acclimation towards plant fitness depends on the dynamics of its regulation. We expect that genetic manipulation to enhance traits such as drought tolerance or yield will most probably succeed through signalling components such as transcription factors or the tuning of sensory systems rather than through direct manipulation of specific physiological traits such as stomatal conductance; for example, the introduction of the gene HaBH4, encoding a transcription factor related to hormonal regulation, has been successful in increasing drought tolerance in crops with only minor trade-offs in the absence of drought (González *et al.*, 2019, 2020). To manipulate traits in this way, we first need to understand how such regulation contributes to yield of crops in the field and to the success of wild plants in specific habitats. This approach can contribute to making science more effective for agriculture, a problem in need of urgent solutions (Passioura, 2020; Sadras *et al.*, 2020).

### Ecological and agricultural implications

Plants have evolved sensory mechanisms that allow the acquisition of information from cues and signals, frequently relying on correlations among environmental variables. Climate change is expected to alter the coupling of environmental variables, changing the information they carry. Global warming is altering the relationship between temperature and length of the photoperiod, with implications for both natural and agricultural systems. For example, such altered environmental correlations are important for winter hardening in trees (e.g. Hänninen and Tanino 2011) and crops (e.g. Peltonen-Sainio *et al.*, 2015). Given that different organisms may rely on different cues for timing of developmental events, indirectly, a decoupling among environmental cues may break the synchrony of behaviour and development altering plant–plant, plant–pollinator, and trophic interactions, in this way changing flows of energy and matter in ecosystems (e.g. Brooker, 2006; Salinari *et al.*, 2006; Deacy *et al.*, 2017; Kharouba *et al.*, 2018).

The proposed model provides a unifying theoretical framework for the study of the ecological role of pre-emptive acclimation in plants, linking environment and plant phenotype across multiple time scales. It has the potential to also contribute to more accurate predictions of the effects of future climate on vegetation.

Conceptual tools to scale molecular understanding to acclimation are also relevant for crop improvement. Current research efforts in plant biology aiming at crop improvement seek to generate more, better, and cheaper genetic and phenotypic data; however, conceptual models of the crop phenotype are lagging (Sadras, 2019). Supporting breeding objectives through the manipulation of the complex signalling pathways involved in metabolic acclimation and regulation processes driven by environmental sensing requires updated models such as the one proposed here. Such regulatory processes can be best understood in relation to the acquisition and use of information. Our model formalizes such an analysis at a high level of abstraction. Less abstract models, specific to pre-emptive acclimation for a given environment and plant species, can be derived from it.

## Abbreviations

ET: evapotranspiration
ET_0_: reference (or potential) evapotranspiration
FR: far-red light
PAR: photosynthetically active radiation
R: red light
VOC: volatile organic compound
